# Building Local Research Capacity for Global Pandemic Preparedness: Lessons from WHO Unity Studies and Their Expansion in India

**DOI:** 10.3390/v18020198

**Published:** 2026-02-01

**Authors:** Yasir Alvi, Farzana Islam, Mohammad Ahmad, Richa Gautam, Aqsa Shaikh, Musharraf Husain, Kartikey Yadav, Mohammad Rashid, Shyambhavee Behera, Nicki L. Boddington, Ashok Basnet, Pushpa Ranjan Wijesinghe

**Affiliations:** 1 Department of Community Medicine, Hamdard Institute of Medical Sciences and Research, New Delhi 110062, India; richa.gautam1@gmail.com (R.G.); emailtoaqsa@gmail.com (A.S.); drkartikeyy@gmail.com (K.Y.); mohammad.rashid465@gmail.com (M.R.); shyambhavee@gmail.com (S.B.); 2 World Health Organization (WHO), New Delhi 110062, India; 3 Department of Surgery, Hamdard Institute of Medical Sciences and Research, New Delhi 110062, India; 4 WHO Health Emergencies Programme, World Health Organization, CH-1211 Geneva, Switzerland; 5 WHO Health Emergencies Programme, South-East Asia Regional Office, New Delhi 110002, India

**Keywords:** pandemic preparedness, Unity studies, Influenza, disease transmission, epidemic, public health action, infection prevention and control

## Abstract

The rapid onset and progression of the COVID-19 pandemic highlighted the critical necessity for standardized, timely epidemiological investigations to generate actionable evidence for public health policy. The World Health Organization (WHO) Global Influenza Surveillance and Response System (GISRS) and Unity Studies and Investigations initiative (Unity Studies) provides the standardized framework to address these critical knowledge gaps. This manuscript reflects upon the Hamdard Institute of Medical Sciences and Research (HIMSR)’s experience as an active site implementing three WHO Unity protocols between 2020 and 2021. We synthesize key findings from the Household Transmission Investigation (HHTI) and the Health Facility Transmission (HCW cohort) studies, detail the operational and analytical complexities addressed through intensive collaboration with WHO HQ, South-East Asia Regional Office (SEARO), and WHO India, and outline the subsequent institutional capacity transmission. Building directly on this established expertise, HIMSR has been designated as the dedicated Nodal network site for the WHO SEAR Unity Studies Network in India, coordinating administration activities of a vast network of national institutes for ongoing pandemic preparedness. This trajectory demonstrates the potential for low- and middle-income country (LMIC) institutions not only to contribute critical evidence during crises but also to transition into resilient national and regional research and surveillance platforms for future pan-respiratory pathogen threats. We detail the essential findings and operational lessons from the COVID-19 pandemic response and elaborate extensively on the strategic implementation plan for the proposed WHO Unity Nodal Network site in India, emphasizing capacity building, standardization, and the integration of research into public health policy.

## 1. Introduction

The advent of Severe Acute Respiratory Syndrome Coronavirus 2 (SARS-CoV-2) initiated the COVID-19 pandemic, creating uncertainty over the virus’s epidemiology and transmissibility properties [1]. To quickly gather evidence-based knowledge and guide public health actions, the World Health Organization (WHO) launched the Investigations and Studies Global Initiative, also known as the Unity Studies [2]. The Unity Studies offer a generic framework for preparedness and readiness for responding to epidemics and pandemics, rapidly adapted from existing protocols (including the Consortium for the Standardization of Influenza Seroepidemiology (CONSISE)), for carrying out standardized investigations essential for the risk assessment of emerging respiratory pathogens [2,3,4]. This standardized architecture guarantees systematic data collection and promotes international comparability, allowing the global community to collaboratively address knowledge gaps [5,6].

Standardized protocols, including the Household Transmission Investigation (HHTI) and the Healthcare Worker (HCW) risk assessment, were crucial for addressing key uncertainties related to transmissibility, severity, and the extent of asymptomatic infection [2,3,4]. This was conducted alongside giving priority to research equity, where these studies supported LMIC serological tests and technical support [2].

The Hamdard Institute of Medical Sciences and Research (HIMSR), New Delhi, is a medical college established in 2011. It provides quality healthcare at an affordable cost supported by the 710 bed Hakeem Abdul Hameed Centenary (HAHC) Hospital, and is well equipped with National Accreditation Board for Testing and Calibration Laboratories (NABL) having adequate biosafety level. A separate Central Research lab is an added feather in the crown of HIMSR. It was one of the sites of WHO’s Unity studies global network during this important time. Between December 2020 and July 2021, the team successfully adapted and completed three different WHO Unity protocols and published six studies in high-index journals [1,7,8,9,10,11]. These were as follows:

1. Household Transmission Investigation (HHTI): A prospective case-ascertained study of COVID-19 transmission dynamics [1,8,10].
2. Health Care Worker (HCW) Cohort Study: An assessment of infection risk factors and prevention measures among healthcare personnel [7,11].
3. COVID-19 Vaccine Effectiveness (VE) Study: A case-control study conducted in collaboration with the Indian Council of Medical Research (ICMR) and WHO [9].

These studies offered timely local data from Delhi, which were helpful during the second wave of COVID-19, possibly driven by the B.1.617.2 “Delta” variant. As a result of the successful implementation of these standardized protocols, coupled with working closely with district and state authorities and WHO offices (HQ, SEARO, and India Country Office) and subsequent academic publications, HIMSR became a significant institution for contributing in generating evidence for national and global pandemic planning. This manuscript discusses the lessons learned from these projects and details HIMSR’s ongoing transition into Nodal network site of the WHO Global Influenza Surveillance and Response System (GISRS) and Unity Studies and Investigations initiative (WHO Unity 2.0) [2,12]. We also tried to describe the programmatic data concerning the operational challenges, capacity strides made, and compilation of the experiences from all three studies at a programmatic level. The goal is to build preparedness, response and resilience against future respiratory threats, in line with the Preparedness and Resilience for Emerging Threats (PRET) framework and the recently accepted WHO Pandemic Agreement [2,12,13].

## 2. The Rationale for Focused Unity Network in India

### 2.1. Indian Context

The Indian context poses complex public health challenges, characterized by high population density, which were significantly exacerbated during the COVID-19 pandemic and its prevention activities. In early 2021, India had the second-highest number of COVID-19 cases in the globe, and New Delhi saw some of the worst spikes [1]. Overcrowding and poor housing conditions can make lockdowns and isolation less effective, which could make it more likely for close contact to spread the virus. The HIMSR HHTI study focused specifically on these dynamics in Delhi’s South and South-East districts, leading to the significant discovery of a high secondary infection rate (SIR) of 44.6% among unvaccinated household contacts, which was much higher than the average for the whole world [1]. The local data also found risk factors that were specific to the situation, such as being female and taking care of the index case. This means that women, who often act as primary caretakers at the household level, had a higher risk. A distinctive finding in the local context was that an increased number of rooms was substantially correlated with secondary infection. This acute demand for clear local epidemiological data happened at the same time as a systemic need in India to improve the ability to gather evidence that is useful for health policies [1,7].

### 2.2. Philosophy and Origin

The operational philosophy of HIMSR’s participation in the Unity Studies initiative is based on the goal of promoting research equity and strengthening pandemic preparedness through standardized operational research [2]. Building on the GISRS platform—which promotes effective collaboration, virus data sharing with the WHO for global risk assessment, and their commitment to a global public health framework for rapid evidence generation—the Unity Studies initiative emerged from the recognized need after the 2009 H1N1 pandemic to standardize methodologies for early investigations of emerging respiratory pathogens [2,6]. This led to a generic preparedness and readiness framework for influenza pandemics, which was rapidly adapted for SARS-CoV-2 in January 2020 [5].

For HIMSR, implementing the Unity protocols meant moving from passive surveillance to systematically investigating transmission dynamics and the whole spectrum of disease, including asymptomatic infection, within closed populations. By adopting WHO’s Unity Studies protocols, the HIMSR team was able to use standardized methods, obtain technical support and funding (including from the WHO Unity Studies program), and use data management system tools, thus reinforcing local research resilience [3,4]. This standardization ensured that the data collected were robust and internationally comparable and also could be used for triangulation and providing global and regional estimates [2,10]. This alignment directly supported the strategic aim of building capacity in LMICs. The strong institutional network was cemented through interaction and guidance from WHO Country Office staff and WHO SEARO, positioning HIMSR as a regional center of expertise [13]. These types of strategic partnership between the WHO and research institutions provided a platform which ensures that future research activities are meticulously organized and primed for swift implementation during future health emergencies, maintaining focus on LMIC capacity building as well as contributing to global risk assessments [2,13].

## 3. Overview of the WHO Unity Platform

The WHO GISRS Unity Studies and Investigations initiative, or Unity Studies, is an innovative way of integrating surveillance techniques and systemic research. This initiative offers a robust global platform for pandemic preparedness, targeting emerging or re-emerging respiratory pathogens [2,13,14]. Unity Studies serve as essential instruments that supplement routine surveillance systems by addressing specific public health inquiries concerning transmissibility, population immunity, and infection/disease severity that routine systems may not efficiently capture [2]. The framework ensures data gathered across diverse settings is comparable and robust for pooled analysis [2,6]. The core of the platform is the collection of standardized template protocols, developed in collaboration with technical partners like CONSISE and the University of Melbourne [2]. Some of the most important sorts of investigations are as follows:

- First Few X (FFX) Cases and Contacts Protocol: For early and rapid data collection on initial cases.
- Household Transmission Investigation (HHTI): A prospective case-ascertained study focusing on transmission in defined household settings.
- Health Worker (HCW) Risk Factor Protocol: A cohort study examining transmission and the effectiveness of Infection Prevention and Control (IPC) measures in healthcare settings.
- Population-based age-stratified sero-epidemiological investigation protocol: Used to measure population immunity/seroprevalence.
- Vaccine Effectiveness (VE) Protocols: For rapid assessment of the effectiveness of vaccines.

**Figure 1:**
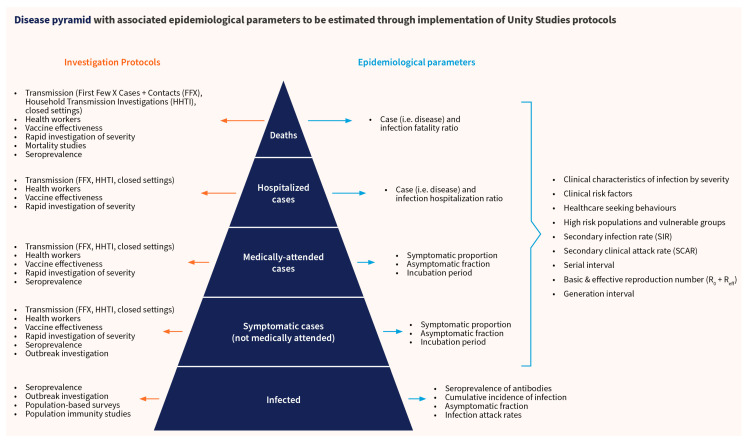
Disease pyramid with associated epidemiological parameters to be estimated through implementation of Unity Studies protocol, Adapted with permission Bergeri et al., 2024 [2,15].

These protocols let researchers estimate multiple epidemiological parameters across the disease pyramid, from asymptomatic infection at the base to disease severity and fatality ratios at the top (Figure 1).

The current Unity Studies 2.0 focuses on sustaining operational readiness through the development of a Global Operational Network of Sites. This network is aligned with the PRET initiative, ensuring readiness is exercised during “peace time” respiratory outbreaks, such as seasonal Influenza and Respiratory Syncytial Virus (RSV) epidemics. HIMSR has formalized its commitment to this network by signing the Terms of Reference (ToR) in December 2024 [13]

## 4. Reflections on Program Design and Implementation

HIMSR successfully implemented and completed three Unity projects during 2020–2021, covering the periods when India’s first wave of COVID-19 was receding and the second wave was peaking.

**Figure 2:**
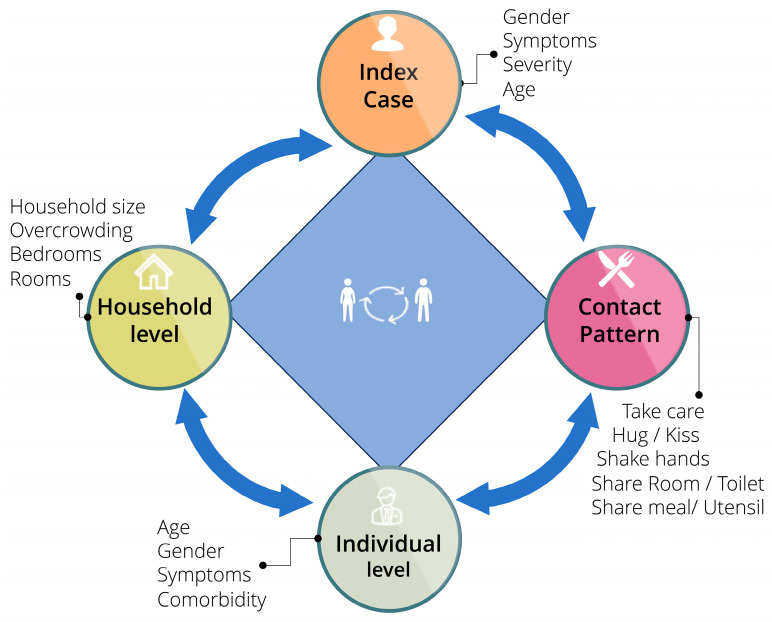
Factors governing transmission dynamics among household members across four dimensions. The transmission of disease among household members is affected by the interaction of four sets of factors, including index case characteristics (age, gender, and symptoms and their severity), household level (household size, number of bedrooms, room for isolation, overcrowding), individual level (age, gender, symptoms, comorbidity), and contact pattern (taking care, hugging, kissing, handshaking, sharing room, toilet, meals, and utensils); Adapted with permission from Islam et al., 2024 [1].

The first project was HHTI [1]. This prospective, case-ascertained study used the WHO Unity HHTI protocol (Version 2.2) [3]. The primary objective was to learn about transmission dynamics and identify associated risk factors of secondary infection among households’ contacts (HHC) of COVID-19 in South and South-East Delhi. The study enrolled 99 index cases and 316 HHCs. The study employed a four-dimensional conceptual framework to examine factors affecting transmission: index case characteristics, household level factors, individual level factors, and contact patterns (Figure 2). Data, along with biological samples (RT-PCR and paired serum samples for serology using WANTAI kits), were collected during four household visits on days 1, 7, 14, and 28 [1].

The key results were (HHTI) as follows:

- High Transmission: The secondary infection rate (SIR) was exceptionally high at 44.6% (95% CI 39.1–50.1). Cumulative secondary attack rate (SAR) was 55.5%, and the household SAR was 54.9%.
- Epidemiological Parameters: Mean serial interval was 7.97 days (±6.66), and the effective reproduction number 1.33 (±1.56) and detectable shedding was 12.49 days (index cases) and 11.84 days (contacts).
- The four dimensions transmission dynamics showed significant associations with secondary infection:
  - Individual Level: Being female (OR 2.13), increasing age (OR 1.01), and symptoms at baseline (OR 3.39) or during follow-up (OR 3.18).
  - Index Case Level: Symptoms during follow-up (OR 6.29) and increasing age (OR 1.03).
  - Household Level: Having more rooms (OR 4.44).
  - Contact Pattern: Taking care of the index case (OR 2.02).

The second project was the Health Care Worker (HCW) Prospective Cohort Study [7]. This study adhered to the WHO Unity protocol for risk assessment among HCWs, identifying risk of disease transmission among those who were exposed to COVID-19 patients at HIMSR and HAHC Hospital [4]. The study was conducted from December 2020 to June 2021 and enrolled 192 HCWs, overcoming initial exclusion criteria against seropositive individuals through a protocol deviation implemented after the national vaccination campaign launched on January 16, 2021. Serology (using WANTAI kits) was collected at baseline and endline (28 days). We found a high (62%) baseline seropositivity, which rose to 77.7% at endline. We also document the Seroconversion Rate at 36.7% overall. Doctors showed the highest rate (63.2%), followed by nurses (42.9%) and paramedics (13.0%). Vaccination has a major risk status for seropositivity at baseline (partial: OR 3.303; full: OR 2.428) and seroconversion (fully vaccinated OR 32.63). High adherence to IPC was generally reported (e.g., 94.7% of nurses used gowns in close contact). However, the study did not find a statistically significant association between adherence to IPC measures (including PPE use and hand hygiene) and seroconversion/titer increase. This finding was likely confounded by the high vaccination rates.

The third project was the Vaccine Effectiveness (VE) case control study [9]. It was a hospital-based, multicentric study with severe COVID-19 patients as the cases and those with COVID-19 negative as the control. The random effects logistic regression model to calculate the adjusted odds ratios (aOR) with a 95% confidence interval (CI) after adjusting for relevant known confounders was used. A total of 1143 cases and 2541 control patients were enrolled across 11 hospitals in India. The VE of complete vaccination was 85% (95% CI: 79–89%) with AZD1222/Covishield and 71% (95% CI: 57–81%) with BBV152/Covaxin [9].

**Challenges of implementing** Unity **Studies at HIMSR:** The study faced operational challenges, which are typical of emergency research. As these protocols were based on global generic Unity protocols and needed to be adopted and implemented at quick pace at the early phase of the COVID-19 pandemic, shortcomings existed. These gaps were later identified during the implementation phase and protocol deviations were necessary. Further, due to variable transmission waves, there were times of declining cases and their subsequent study enrollment, an retrospective enrolment strategies were implemented and added as protocol deviation. Calculating precise parameters like the incubation period (IP), serial interval, and Basic Reproduction Number was complicated by continuous daily household interaction, making the exact date of exposure difficult to ascertain. Furthermore, analyzing co-primary cases increased the complexity of estimating secondary infection rates. In the HCW study, we faced the issue of high baseline non-response and significant loss to follow-up. The unpredicted and early vaccination campaign for HCW introduced significant confounding variables, preventing clear immunological differentiation between infection-induced and vaccine-induced seroconversion. The VE study at HIMSR too faced severe shortages of case recruitment during the study period. As a measure, the shortage was compensated by additional participant recruitments at other study sites within this multicentric study.

Programmatic Strengths: The projects benefited immensely from strong WHO support for protocol finalization, financial oversight, monitoring, analysis, and dissemination. State health administration and district health authorities too extended technical support and access to district contact tracing data for comprehensive data management and contact tracing, which were some of the major pillars of successful implementation.

## 5. Reflections on the Strides Made and Challenges Faced by the Program

The three COVID-19 Unity Studies conducted by the HIMSR team had both significant achievements and inherent difficulties in epidemiological research. The study team held multiple brainstorming and problem-solving sessions during and after the studies to understand the underlying causes of these challenges and to identify potential solutions, all of which are discussed below.

**Strides Made (Individual and Collective Capacity)**

1. *Generation of High-Quality, Contextual Evidence:* HIMSR successfully delivered a comprehensive understanding of the COVID-19 epidemic specific to Delhi, India during a period of high transmission. This evidence included important estimates such as the high Household SAR (54.9%) and risk factors (e.g., caregiving, gender, dwelling space). This strict adherence to standard methods made it possible for local knowledge to be shared and collaborated with global networks.
2. *Strengthening the Validity of Unity Protocols in LMICs:* The success validated the operational utility of complex, multi-visit, longitudinal WHO Unity protocols (HHTI, HCW) in India, which included sophisticated data collection and sequential biological sampling (RT-PCR and serology).
3. *Advanced Technical and Analytical Capacity:* The WHO trainings provided the team the opportunity to learn sophisticated skills in planning and analysis, including calculating complex parameters.
4. *Established Networking and Global Visibility:* Strong operational partnerships were built with WHO and with national institutes. This infrastructure, facilitated by key WHO personnel, positioned HIMSR as a regional center of expertise and contributed to global standardization efforts.
5. *Multidisciplinary Workforce Development:* The projects required the recruitment and training of specialized staff (trained healthcare workers, phlebotomists, data managers), enhancing local capacity for rapid deployment research.

**Challenges Faced**

1. *Methodological Ambiguity in Key Parameter Calculation:* As discussed earlier, it was hard to obtain accurate estimates of parameters like the incubation period and the serial interval due to continuous daily household exposure and the non-specificity of symptomatic definitions.
2. *Sampling and Follow-up Biases:* The use of convenience sampling (in HHTI and HCW cohort studies) for index case recruitment and the high rates of non-response and loss to follow-up may have led to possible selection bias, validity, questionable generalizability and lower statistical accuracy. The exclusion of hospitalized participation in the HHTI study resulted in the cohort omitting the most severely affected segment of the population, but was essential exclusion criteria as per HHTI Unity protocol.
3. *Low enrolments during inter-peak transmission:* Our studies (HHTI and HCW cohort studies) spanned through many transmission cycles, with very high infection transmission in the early phase and with new strain and dropping to a low level in between. With low positivity during inter-peak transmission combined with high rates of non-response, we had to adapt protocol deviation and enrolled participants retrospectively. In HHTI, to help with enrolment, we also expanded our target population, involved other districts in Delhi, and worked with their district health authorities for data sharing. The VE trial study also faced low enrolment and other partner sites stepped in to compensate the low enrolment at HIMSR.
4. *Confounding by Vaccination and IPC Measures:* The unexpected early rollout of the mass HCW vaccination campaign in the middle of the study severely confounded the results, especially in the HCW cohort. It became impossible to immunologically differentiate between infection-induced versus vaccine-elicited immune responses. Furthermore, despite reporting high adherence to PPE and robust institutional IPC measures, in the HCW study, we failed to find a significant protective association between individual IPC measures and reduced seroconversion risk.
5. *Resource and Logistics Constraints:* Administrative delays impacted timelines. The need to adapt protocols under time, budget, and human resource constraints led to limitations in ancillary objectives, such as deferring genotyping studies, which would have been useful for understanding variant transmission.

## 6. Proposed Unity Network for HHTI in India

The foundational capacity built by HIMSR during the COVID-19 pandemic response facilitated its transition and positioned it as a regional network site within the WHO Unity Studies Network (WHO Unity 2.0) [15]. This strategic move formalizes HIMSR’s commitment to resilient pandemic preparedness, aligning with the global goals of the PRET and WHOs Mosaic respiratory surveillance framework [2]. This initiative is centered on exercising standardized protocols during inter-pandemic “peace time” with quick action during novel disease outbreaks [2,16]. Below, we have detailed the network’s plans for structure, methodology, network expansion, and capacity building, emphasizing the transition from local expertise to a national resource.

**A. Strategic Alignment and Core Objectives of the Unity Studies network site at HIMSR:**

The HIMSR network site is strategically located in the WHO South-East Asia Regional Office (SEARO) chapter of the Unity Studies network, where it is responsible for carrying out the standardized Unity Studies. This network aims to increase the evidence-based knowledge for action, promote international comparability of epidemiological investigations, and support national public health and social measures [15]. The Unity Studies 2.0 initiative requires the establishment of a global operational network of sites prepared for the rapid assessment of novel respiratory viruses [2]. HIMSR solidified this commitment by having its senior management sign the Terms of Reference (ToR) with the WHO in December 2024 [13].

HIMSR’s role as a regional network site is primary in systematically implementing the rigorous methodology and exercising investigations at Nodal and satellite sites during inter-pandemics (e.g., seasonal influenza) to maintain operational readiness [2,15]. This aligns with the “Mosaic Respiratory Surveillance Framework,” where Unity Studies act as specialized tools that enhance routine systems [2,14]. In the proposed network, HIMSR will function as a Unity Studies network site, responsible for liaising with the WHO, coordinating administration activities, conducting training, consolidating reporting, and carrying out the mandatory tasks of adapting and exercising one of the Unity protocols during the inter-pandemic period. This is being piloted by HIMSR’s adaptation of HHTI in an ongoing study [16]. This “exercising” phase is crucial for strengthening preparedness and rapid response mechanisms. This comprehensive approach ensures that HIMSR generates essential new knowledge and integrates existing fragmented regional evidence to guide policy, vaccination strategies, and public health capacity thus ensuring preparedness for a future pandemic [13,17].

**B. Exercising Readiness—Primary Research Focus for 2025:** HHTI on Influenza and RSV

The immediate priority is exercising the core HHTI protocol adapted for Influenza A, Influenza B, and Respiratory Syncytial Virus (RSV) among Delhi residents, commencing in 2025 [13,16,17]. This is justified by the rising burden of these viruses and aligns with WHO guidance and HIMSR’s proven institutional expertise in respiratory pathogen research [17]. Given the deteriorating air quality in India’s capital, this research may also uncover the extent of undocumented respiratory illnesses [18].

**C. Protocol Adaptation and Scope Limitation:** Based on lessons of resource constraints during the COVID-19 projects, the ongoing HHTI study is designated as a pilot study with strict scope limitations [17]:

- Pathogen: Focus shifted from a novel pathogen to Influenza A and B and RSV. Testing utilizes RT-PCR via the GeneXpert system (Xpert Xpress Flu/RSV test cartridges) for the simultaneous detection and differentiation of Influenza A and B and RSV viral RNA, utilizing in-house accredited PCR facilities
- Sampling Strategy (Pilot Phase—Oct to Nov 2025): The initial pilot is strictly constrained by time, budget, and human resources. It targets a small sample size of five index cases and all their household contacts to validate the adapted methodology before scaling.
- Data Collection Schedule: Simplified sampling schedule with only nasal samples on day 1 and day 7, with a symptom diary kept until day 14. Serological testing is not planned for this phase.
- Deferred Objectives: Advanced analyses, including the calculation of incubation period, serial interval, R0, and genomic analysis, are deliberately excluded from the pilot phase due to time, budget, and size constraints. The focus is limited to achievable primary outcomes like SIR and SCAR, thus a part of scope limitation.

**D. Network Expansion and Collaborative Expertise (**Satellite Sites**)**

As a Unity Studies network site, HIMSR’s responsibility extends to peripheral institutions or “Satellite Sites” to strengthen and expand the Unity Studies Network across India. This network expansion is essential for fulfilling the strategic goal of achieving wide geographical and demographic representation, a critical component of global preparedness [2].

1. Proposed Satellite Sites: The satellite sites will perform the program tasks of exercising Unity protocol, in consultation with the Nodal network site. HIMSR has proposed and invited several premier academic and research institutions across India as potential satellite sites for coordinating future HHTI exercises. These institutions demonstrated their commitment and research capacity during the COVID-19 Unity Studies and will utilize their experiences.

**Figure 3:**
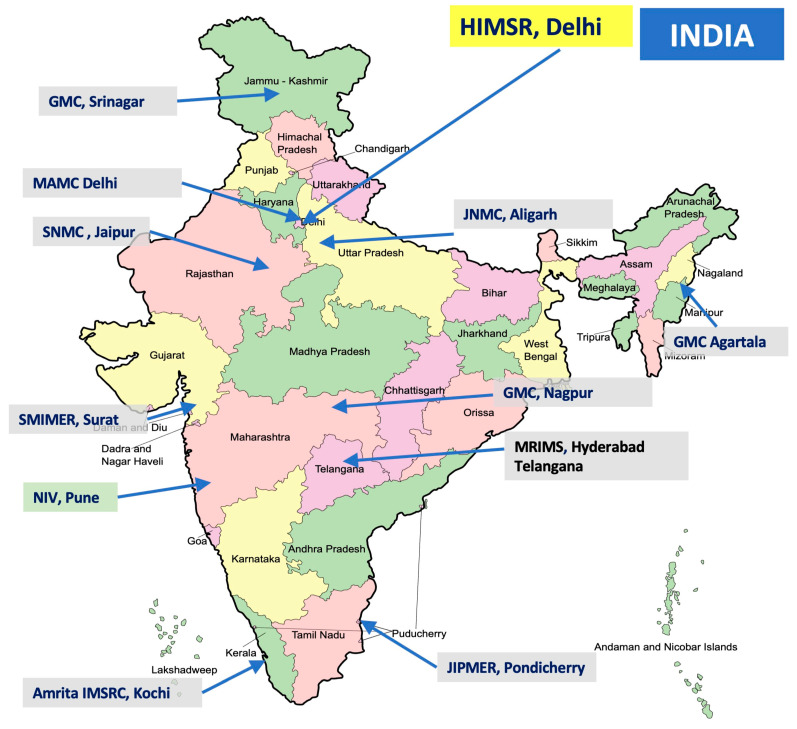
Medical institutes proposed for inclusion in the HHTI Unity Network, India. Footnote: Hamdard Institute of Medical Sciences and Research (HIMSR), New Delhi India; Government Medical College, Srinagar; Maulana Azad Medical College, Delhi; Amrita Institute of Medical Sciences, Kochi; Government Medical College, Agartala; Malla Reddy Institute of Medical Sciences (MRIMS), Hyderabad; SMIMER Medical College, Surat; Government Medical College, Nagpur; Jawaharlal Nehru Medical College, Aligarh; S.N. Medical College, Jaipur; Jawaharlal Nehru Institute of Post Graduate Medical Education & Research (JIPMER), Pondicherry, and ICMR- National Institute of Virology (NIV), Pune. Sites highlighted with Grey color are proposed satellite sites and with green color is WHO reference lab.

This distributed network strategy ensures the Nodal site can rapidly scale up surveillance efforts in diverse geographical and health system contexts when a novel pathogen emerges (Figure 3).

2. Nodal network site Responsibilities and Requirements: To ensure the effective function of the network, all Unity Studies Network Sites, including HIMSR, must adhere to stringent duties outlined in the Terms of Reference (ToR). These duties include the following [19]:

- Designated Focal Point: The site must have a designated site Principal Investigator (PI)/Focal Point to coordinate investigations and liaise with the WHO Country Office.
- Technical Participation: Sites must participate in relevant conference calls to identify and collaboratively solve issues early in the investigation phase, promoting international collaboration and sharing preliminary global findings.
- Research Translation: By exercising standardized protocols and establishing multi-institutional collaborations, all Unity Studies Network Sites are strengthening the research–policy–practice interface, promoting the generation of evidence that can influence policy, thereby addressing a critical need in LMICs where translation supporting structures are traditionally weak.
- Knowledge Sharing and Laboratory Links: The network aims to utilize the isolated expertise across India by creating a platform for shared learning and joint mentorship among participating sites. Another important benefit is that sites would have access to a designated a national-level laboratory which is a WHO Collaborating Center and National Influenza Centre of India and WHO Global H5 reference lab under GISRS (NIV, Pune) [19]. In addition, the sites must be willing and prepared to share isolates and specimens with the WHO Collaborating Centers.
- Data Analytics and Expertise: Sites require access to a dedicated data analytics team to perform rapid analysis. The HIMSR team’s analytical capacity, supported by WHO India and WHO HQ, involving complex analysis, is foundational to fulfilling this requirement.

3. Benefits of Participation and Equity: Participation in the Unity Network provides significant benefits, particularly for LMICs [2,15]. Sites are recognized globally as a center of expertise, enabling the unlocking and mobilization of knowledge across geographic and disciplinary boundaries. The network facilitates capacity building by offering skills training in study implementation, laboratory testing, data management, and statistical/modeling analyses [2]. The overall initiative promotes research equity by ensuring that high-quality, standardized research is feasible and supported in LMICs, reducing the historical publication bias towards high-income countries. The WHO provides access to standardized and harmonized tools, such as generic field questionnaires, data collection tools, and data analysis scripts, promoting interoperability among sites [16].

**E. Bridging Research and Systems Capacity**

The function of the HIMSR network site extends beyond data collection to ensure the effective translation of research findings into policy. This aligns with wider national acknowledgment of the importance of building Health Systems Research.

1. Science Translation for Decision-Makers: The role of government, health authorities, and policymakers in sustained engagement within the Unity Studies Network is centered on transforming high-quality research into actionable public health policy and securing operational support. Timely dissemination of findings is crucial to update guidance and inform local, national, and international public health responses. The ultimate goal is for the standardized HHTI data to be available to decision-makers and key stakeholders to inform timely policy-relevant decisions [16]. The standardized HHTI data must be made available to decision-makers and key stakeholders to enable them to inform timely, policy-relevant decisions. This translational goal ensures that timely dissemination of findings updates guidance and informs local, national, and international public health responses. To facilitate this, the Unity protocols provide toolkits and training on how to communicate with different audiences and address policy-makers’ questions.

2. Addressing Methodological Rigor for Aggregation: For the data collected by the HIMSR and its network to be pooled and effectively contribute to global policy [2,10], methodological rigor must be consistently high [6]. The WHO Unity team developed specialized tools, such as the HHTI Critical Appraisal Checklist, consisting of 12 questions covering 10 aspects like context, household definition, and follow-up quality, to assess potential for bias and suitability for aggregation [6]. The HIMSR commits to ensuring its implementation meets these high standards.

3. Sustained Engagement: The establishment of the Regional network site contributes to overcoming the historical insularity of expertise. By fostering formal collaborations between diverse institutions, the network site promotes a necessary collective level of capacity building. This mechanism helps ensure that the generated evidence is locally relevant and improves the research–policy–practice interfaces, which are often traditionally weak in LMICs. The lessons learned from other Indian capacity-building initiatives, such as the need to align research topics with policy-makers’ interests for better buy-in, are intrinsically integrated into the network site’s mission to provide timely, actionable data during future outbreaks [13].

**F. Budgetary and Ethics Considerations**

Sustained operation requires robust financial planning. With the changing health financing global scenario and reduction in WHO financial support to the LMIC, the need for internal financial resource generation and co-funding by the network institutions for sustainable project implementation highlighted the scale of resources required. With the previous experiences during the COVID-19 HHTI study, the largest cost components were for testing samples, followed by costs for Infection Prevention and Control (IPC) materials, transport, and personnel costs. The Unity Studies network site operates on catalytic WHO support, emphasizing co-funding by HIMSR to ensure sustainability and country ownership. Furthermore, the WHO commits to providing support for protocol finalization, financial oversight, monitoring, analysis, and dissemination of data.

The successful conduct of the HHTI requires meticulous adherence to Infection Prevention and Control (IPC) measures, necessitating consistent funding for PPE and waste disposal. Adhering to ethical standards, including obtaining written informed consent in the local language, adequate data management and maintaining participant confidentiality through assigned identification numbers are also resource intensive and non-negotiable. Furthermore, the study must undergo rigorous ethical clearance processes, including approval from local (HIMSR) and WHO SEARO Ethics Review Committees for the study. Focusing on the principle of justice, the operational philosophy of the Unity Studies Network is based upon the global objective of promoting research equity.

## 7. Conclusions

The HIMSR team’s rigorous execution of three distinct WHO Unity protocols between 2020 and 2021—HHTI, HCW cohort, and VE study—marked a significant contribution to both national and global understanding of SARS-CoV-2 dynamics, validating the critical role of standardized operational research in pandemic response. Key findings and vital epidemiological data provided immediate, context-specific evidence necessary for refining and implementing effective local public health measures.

The program effectively developed robust technical and human resource capacities, especially through close collaboration and networking with the WHO (HQ, SEARO, India Country Office) and utilization of global tools. Critical lessons learned included the necessity of managing confounding variables and addressing the methodological complexity of estimating transmission parameters in high-contact settings.

This experience provided the necessary operational capacity and intellectual foundation for HIMSR’s designation as a WHO Unity Studies Network Site. The strategic transition focuses on sustaining preparedness through the 2025 HHTI pilot study on endemic respiratory pathogens (Influenza/RSV). By rigorously adhering to standardized protocols, focusing on practical capacity building, and actively expanding a national network of collaborating institutions, the HIMSR ensures that India develops a resilient operational research infrastructure capable of rapidly providing standardized, high-quality, and contextually relevant data to guide effective public health responses against future respiratory threats.

## Data Availability

No new data were created or analyzed in this study. Data sharing is not applicable to this article.

## Abbreviations

CI	Confidence Interval.
CONSISE	Consortium for the Standardization of Influenza Seroepidemiology.
COVID-19	Coronavirus Disease 2019.
FFX	First Few X Cases and Contacts Protocol.
GISRS	Global Influenza Surveillance and Response System
HAHC	Hakeem Abdul Hameed Centenary Hospital.
HCW	Health Care Worker or Health Worker.
HHC	Household Contacts.
HIMSR	Hamdard Institute of Medical Sciences and Research.
HHTI	Household Transmission Investigation.
ICMR	Indian Council of Medical Research.
IPC	Infection Prevention and Control.
IP	Incubation Period.
LMIC	Low- and Middle-income Country
NABL	National Accreditation Board for Testing and Calibration Laboratories
OR	Odds Ratio.
PI	Principal Investigator.
PPE	Personal Protective Equipment.
PRET	Planned Preparedness and Resilience for Emerging Threats.
RSV	Respiratory Syncytial Virus.
RT-PCR	Reverse Transcription Polymerase Chain Reaction.
SAR	Secondary Attack Rate.
SARS-CoV-2	Severe Acute Respiratory Syndrome Coronavirus 2.
SEARO	South-East Asia Regional Office.
SIR	Secondary Infection Rate.
ToR	Terms of Reference.
VE	Vaccine Effectiveness.
WHO	World Health Organization.
WHO HQ	WHO Headquarters
